# Adolescent Health and Well-Being: Background and Methodology for Review of Potential Interventions

**DOI:** 10.1016/j.jadohealth.2016.07.023

**Published:** 2016-10

**Authors:** Rehana A. Salam, Jai K. Das, Zohra S. Lassi, Zulfiqar A. Bhutta

**Affiliations:** a Division of Women and Child Health, Aga Khan University, Karachi, Pakistan; b Robinson Research Institute, University of Adelaide, Adelaide, Australia; c Centre for Global Child Health, The Hospital for Sick Children, Toronto, Canada; d Center of Excellence in Women and Child Health, The Aga Khan University, Karachi, Pakistan

**Keywords:** Adolescent health, Young adults, Youth, Delivery platforms

## Abstract

Owing to child survival initiatives around the world in the 1970s and 1980s, a dramatic rise in the population of adolescents has been seen, especially in the developing countries. A quarter of world's population in 2012 comprised adolescents and young adults; of these, 90% lived in low- and middle-income countries. More recently, there has been a consensus on investing in adolescent health and development for the success of post-2015 developmental agenda. In this series of articles, we aimed to assess various interventions identified in our conceptual framework to evaluate their effectiveness in improving adolescent health. We took a systematic approach to consolidate the existing evidence. This article is an introductory article detailing the background, conceptual framework, and methodology used for synthesizing evidence, followed by seven articles summarizing evidence on interventions for sexual/reproductive health, nutrition, immunization, mental health, substance abuse, and accidents/injury. The concluding article of the series summarizes the findings of the all the previous articles in the series and the relevance of the evidence for action in the post-2015 Millennium Development Goals era along with evidence gaps and future research priorities.

Adolescence is a critical age group as this is a period to develop specific expertise and hone individual skills to enter the mainstream workforce and contribute to the economic productivity. It is also a period when major changes in health and health-related behaviors such as smoking and substance abuse, unsafe sexual practices, poor eating, and lack of exercise occur, which may substantially impact health outcomes in later life. Due to the success of child survival initiatives over the last few decades, there has been a dramatic rise in the population of adolescents especially in low- and middle-income countries (LMICs) [1]. A quarter of world's population in 2012 (1.8 billion) comprised adolescents and young adults (10–24 years) [2]; of these, 90% lived in LMICs [3]. It is expected that the proportion of the world's young population, particularly in Africa, will rise from 18% in 2012 to 28% in 2040 while the proportion in all other regions of the world will eventually decline [3].

There is an unacceptable rate of mortality among adolescents, as an estimated 1.3 million adolescents died in 2012; 70% of these deaths occurred in Africa and Southeast Asia [4]. Unintentional injuries such as road traffic accidents and drowning are the leading causes of death in adolescents, while suicide, violence, infectious diseases, and teenage pregnancy are other important causes of mortality in this period [4]. An estimated 330 adolescents die every day of road traffic accidents while 180 adolescents die every day from interpersonal violence [5]. Among females aged 15–19 years, pregnancy-related deaths are the second leading cause of death after self-harm while road traffic accidents and interpersonal violence are the main causes of death among males in this age group [6]. The odds of dying during youth are almost two times higher in South Asia and four times higher in Sub-Saharan Africa than those in other regions [4]. Besides mortality, adolescents are also at risk of many nonfatal diseases and conditions that contribute to years lost to disability and disability-adjusted life years (DALYs) burden. Neuropsychiatric disorders, unintentional injuries, and infectious and parasitic diseases contribute to an estimated 70% of the years lost to disabilities for 10- to 24-year-olds. The main risk factors for incident DALYs in 10- to 24-year-olds include alcohol, unsafe sex, iron deficiency, and illicit drug use [7], [8]. In every region of the world, impoverished, poorly educated and rural adolescents are more likely to be adversely affected than their wealthier, urban and educated counterparts. Moreover, variations have also been observed between males and females, as 52% of deaths in male adolescents are attributed to violence while these attribute to 30% of deaths among female adolescents [9].

Globally, around 16 million babies are born to adolescent girls between the ages of 15 and 19 years [10]. Although rates of births among adolescent girls have declined in all regions since 1990 but still remain high in Africa, Asia, Latin America, and Caribbean. Nearly 50% of all women aged 20–24 years in Asia and Africa are married by the age of 18 years, placing them at a higher risk for early pregnancy, maternal disability, and death [10], [11]. Child marriage is also common in the Middle Eastern countries such as Yemen and Palestine, where about half of young people under the age of 18 years are married [12]. Every year, around 10% of adolescent girls in LMICs give birth, compared to <2% in high-income countries (HICs). Girls under the age of 15 years account for 2 million of the annual total 7.3 million new adolescent mothers, and this could rise to 3 million a year in 2030 if the same rate persists [11]. Pregnancy in adolescence is associated with greater risk to the mother and newborn—including anemia, mortality, stillbirths, and prematurity—especially since the adolescent girls are not yet physically mature themselves [13]. In many contexts, their situation is further complicated by a number of factors including poverty, lack of education and employment, restricted access to care, weak health systems, abuse, unplanned or unwanted pregnancies, and the absence of autonomy or support in their social arrangements.

Adolescence and young adulthood accord with key changes in health and its determinants later in life. The Lancet Adolescent Health series in 2012 reported that adolescents are more exposed to substance abuse, sexually transmitted infections, and other risks than in the past, in addition to facing other emerging challenges such as social media [2], [9], [14], [15]. Adolescence is also an optimal time to target health-related behaviors, as the interventions/behaviors will have more time to take effect and thus will maximize the impact on enhancing an individual's health in the years ahead. Recently, there has been a growing interest in adolescent's nutrition in LMICs as a means to improve the health of women and children. The World Health Organization organized a study group for adolescent health and development along with United Nations Children's Emergency Fund and United Nations Framework for Population Activities in 1995 [2], [14], [15], [16]. More recently, there has been consensus on investing in adolescent health and development for the success of post-2015 developmental agenda [17]. The United Nations' reports that with such large numbers of young people, it is imperative that they should be given the economic and social power as well as the right to a healthy life to handle the future and their own lives. There has been an increased focus on adolescent health with the launch of a Lancet commission on adolescent health and well-being involving a network of academics, policy makers, practitioners, and young health advocates with broad expertise in adolescent health. This commission outlines the opportunities and challenges for investment at both country and global levels [18].

The purpose of this extensive review of eight articles [19], [20], [21] (Faqqah A et al; Salam RA et al; Arshad A et al; Das JK et al, unpublished) was to build upon the existing work in adolescent health and development, by synthesizing the available information to determine the effectiveness of various evidence-based interventions targeting adolescents and to list a set of interventions which are proven to be effective and could be recommended for scale-up in countries. This would aid the growing focus on adolescent health and well-being and help set priorities to achieve the global targets to improve adolescent health.

### Domains of adolescent health—a conceptual framework

The newly developed agenda for Sustainable Development 2030 has recognized a need for greater accountability especially for the Global Strategy for Women's, Children's and Adolescents' Health [22]. It has also called for increased participatory frameworks across a range of areas relevant to young people including infections, noncommunicable disease risks, obesity, women's health, mental health, and nutrition [18], [22]. We developed a conceptual framework through existing conceptual frameworks [2], [23] and consultations and deliberations with the global experts in the field of adolescent heath (Toronto, February 2014), and based on the recommendations, we identified a set of interventions to be incorporated in our review process. The interventions were chosen from the existing work on the basis of proven and potential effectiveness to improve adolescent health outcomes and access to primary health care and commodities for adolescents [2], [9], [14], [15]. Our focus was on adolescent age group, defined as adolescents aged 10–19 years, however, since many studies targeted adolescents along with the youths (aged 15–24 years), we expanded our scope to include interventions for adolescents and youth and reported disaggregated adolescent data, where possible.

Various individual and general risk factors throughout the life cycle can have implications at any stage of the life cycle. The various stages of the life are not independent of each other and impacts early in life are carried to the next stage of life while some can also have intergenerational effects. We will not divulge further into this as the purpose of this series of articles was to review the potential interventions which could alleviate the risk factors of the adolescent age group only and impact quality of life thereon. Figure 1 shows the conceptual framework focusing on adolescent-specific interventions to guide our review. In order to organize the existing body of knowledge on adolescent health, our conceptual framework revolves around risk factors, potential interventions to prevent and manage risk factors, and outputs and impacts at individual, community, and societal levels.

#### Risk factors

We focused on risk factors including risky sexual behaviors, unintended pregnancies, violence, risky driving (including speeding and drunk driving), undernutrition, obesity, infections, and mental health risks. Adolescent girls are two to five times more likely to die from pregnancy-related causes than women aged 20–29 years [24]. Girls younger than 19 years have a 50% increased risk of stillbirths and neonatal deaths, as well as an increased risk for preterm birth, low birth weight, and asphyxia [25]. These health risks further increase for girls who become pregnant earlier than the age of 15 years and are somewhat reduced for older adolescents aged 18–19 years [26], [27], [28], [29]. Adolescent nutrition is also a crucial factor for proper growth and development as it is a prerequisite for achieving full growth potential; failure to achieve optimal nutrition may lead to delayed and stunted linear growth [30]. Over the last two decades, increasing rates of overweight and obesity among children and adolescents have been seen in many countries. Malnutrition in children and adolescents, especially among females, leads to maternal and newborn complications. Many LMICs now bear a double burden of nutritional disorders due to the emerging issue of overweight and obesity along with the existing high rates of undernutrition and other micronutrient deficiencies [31], [32]. Globally, childhood obesity rates continue to rise in LMICs while in HICs, they are gradually plateauing [33], [34]. Childhood overweight is associated with multiple immediate and long-term risks including raised cholesterol, triglycerides, and glucose; type 2 diabetes, high blood pressure, and adult obesity and its associated consequences [35], [36].

Smoking, drugs, and alcohol use are also a significant health concern among adolescents. Alcohol use is a major risk factor and accounts for 7% of the DALYs among 10- to 24-year-olds. Most regular smokers initiate smoking before the age of 20 years and develop the habit over a lifetime after they become addicted. Drug abuse is a contributing factor for unemployment, poor health, accidents, suicide, and mental illness [37], [38]. In adolescents, intravenous drug use and unsafe sexual practices increase the risk for HIV infection. More than 2 million adolescents are living with HIV and millions more are at risk of infection [39]. Furthermore, many young people do not know their HIV status, and it is estimated that in Sub-Saharan Africa, only 10% of young men and 15% of young women (15–24 years) are aware of their HIV status [39]. Infectious diseases have been an area of focus among children, but these are also prevalent in the adolescent age group. Sexually active adolescents have the highest rates of prevalent and incident human papillomavirus infections [40]. Many mental health problems also emerge in adolescence with depression bearing the highest burden of DALYs among young people [4].

#### Interventions

We included a range of potential interventions including (1) sexual and reproductive health interventions (sex education, interventions to prevent unintended and teenage pregnancies, treatment and management of sexually transmitted infections, dating violence prevention, interventions to prevent female genital mutilation, genetic counseling and premarital counseling); (2) nutrition interventions (interventions to promote healthy nutrition, micronutrient supplementation, nutrition in pregnant adolescents, prevention and management of obesity); (3) infections and immunizations (interventions to improve coverage of vaccine for human papillomavirus; measles, mumps, and rubella; and varicella); (4) mental health interventions (pharmacotherapy, psychological and cognitive therapies for depression and anxiety and interventions for suicide and suicidal behaviors); (5) substance abuse (interventions for prevention and rehabilitation of tobacco, alcohol and drug abuse); (6) injury prevention interventions (education for preventing dog bite, use of helmet, violence prevention, and accident prevention); and (7) interventions for adolescents living with chronic diseases.

#### Outputs

Outputs included immediate and direct results achieved immediately after implementing an intervention, and these included access to sexual health services, knowledge of sexually transmitted infections, improved dietary behavior, improved physical activity, increased immunization uptake, access to mental health services, delivery of suicide preventive services, awareness of substance abuse, reduction in mental health risk factors, reduction in negative behavior, and increased life and livelihood skills.

#### Outcomes and impacts

Investments in adolescent health and well-being bring a triple dividend of benefits now, into future adult life, and for the next generation of children [18]. Tackling preventable and treatable adolescent health problems including infectious diseases, undernutrition, sexual and reproductive health, injury, and violence will bring huge social and economic benefits and is highlighted as a key to converge countries by 2030 [18]. We included outcomes and impacts at the individual, community, and societal levels including improved health, better adult life, work productivity, improved family and immediate community, economic growth, and national progress.

## Methods

Our aim was to look at the holistic evidence around the interventions identified in our conceptual framework for which we took a systematic approach to consolidate the existing evidence through the following three methodologies in the order of our priority:

1. Overview of systematic reviews: We conducted an overview of systematic reviews for interventions where recent systematic reviews existed;
2. Updating existing reviews: We updated the existing systematic reviews if the existing review only included evidence before 2011;
3. De novo review: For interventions where no reviews existed, we conducted primary literature search followed by a systematic review of existing literature.

Our primary focus was on interventions targeting adolescents from both LMICs and HICs and where possible report segregated impact of the selected interventions in various settings. Our aim was to report data for adolescent age group where available; however, for certain areas where we could not desegregate the data for adolescents and youth age groups, we have reported the combined results for adolescents and youth as representative of the population of interest. We did not apply any language restriction in our search. The methodology applied for each of the above three approaches are described in the following sections:

### Overview of systematic reviews

We conducted an overview of systematic reviews for interventions where recent systematic reviews existed. We considered all available systematic reviews published between January 2011 and December 2014 on the interventions to improve adolescent sexual and reproductive health, nutrition, immunization, mental health, chronic diseases, substance abuse, and injury prevention. Our priority was to select existing recent Cochrane and non-Cochrane systematic reviews of randomized or nonrandomized controlled trials, which fully or partly addressed the interventions for improving adolescent health. Detailed examination of cross-references and bibliographies of available data and publications to identify additional sources of information was also done. A broad search strategy was used that included a combination of appropriate keywords, medical subject heading, and free text terms. Search was conducted in the Cochrane Library, Medline, and PubMed to identify recent systematic reviews on the predefined interventions in our conceptual framework. The project team set up a triage process with standardized criteria for primary screening. The abstracts (and the full sources where abstracts are not available) were screened by two abstractors to identify systematic reviews adhering to our objectives. Any disagreements on selection of reviews between these two primary abstractors were resolved by the third reviewer. After retrieval of the full texts of all the included reviews, data from each review were abstracted independently and in duplicate into a standardized form. Information was extracted on (1) the characteristics of included studies; (2) description of methods, participants, interventions, and outcomes; (3) measurement of treatment effects; (4) methodological issues; and (5) risk of bias tool. We extracted pooled effect size for the outcomes of interest with 95% confidence intervals (CIs). We assessed and reported the quality of included reviews using the 11-point assessment of the methodological quality of systematic reviews criteria (Table 1) [41].

### Update existing reviews

We updated the existing systematic reviews only if the most recent review on a specific intervention was conducted before December 2011. For updating the existing reviews, we adopted the same methodology and search strategy mentioned in the identified existing review to update the search and find all the relevant studies after the last search date of the existing review. After retrieval of the full texts of all the articles that met the inclusion/exclusion criteria, data from each study were abstracted independently and in duplicate into a standardized form. Information was extracted on study design, geographical setting, intervention type and description, mode of delivery, and outcomes assessed. We then updated the estimates of reported outcomes by pooling the evidence from the new studies identified in the updated search and reported new effect size for the outcomes of interest with 95% CIs. We then assessed and reported the quality of included reviews using the 11-point assessment of the methodological quality of systematic reviews criteria (Table 1) [41].

### Conduct de novo review

We conducted de novo reviews of available evidence for interventions where no reviews existed. We considered all available existing randomized, quasirandomized, and before/after studies on the identified adolescent health interventions. The following principal sources of electronic reference libraries were searched to access available data: the Cochrane Library, Medline, PubMed, Popline, LILACS, CINAHL, Embase, World Bank's JOLIS search engine, CAB Abstracts, British Library for Development Studies at IDS, the World Health Organization regional databases, Google, and Google Scholar. Detailed examination of cross-references and bibliographies of available data and publications to identify additional sources of information was also performed. A broad search strategy was used for each review that included a combination of appropriate keywords, indexing, and free text terms. The titles and abstracts of all studies identified were screened independently by two reviewers for relevance and matching. After retrieval of the full texts of all the studies that met the inclusion criteria, data from each study were abstracted independently and in duplicate into a standardized form containing study design, geographical setting, intervention type and description, mode of delivery, and outcomes assessed.

We conducted a meta-analysis of individual studies and reported pooled statistics as the relative risk for categorical variables and standard mean difference for continuous variables between the experimental and control groups with 95% CIs. The pooled statistics were reported using Mantel–Haenszel pooled method or DerSimonian–Laird method where there was an unexplained heterogeneity. All analyses were conducted using Review Manager, version 5.3 (Cochrane Collaboration, London, United Kingdom), which is a freely downloadable software used for conducting meta-analysis and present the results graphically [19]. The level of attrition was noted for each study, and its impact on the overall assessment of treatment effect was explored by using sensitivity analysis. For all outcomes, we carried out the analysis, as far as possible, on an intention-to-treat basis. Heterogeneity was quantified by χ^2^ and *I*^2^; a low *p* value (<.1) or a large chi-square statistic relative to its degree of freedom and *I*^2^ values >50% were taken as substantial and high heterogeneity. In situations of high heterogeneity, causes were explored by sensitivity analysis and random effect models were used. Our major comparison was the intervention of interest versus standard or routine care, but wherever data permitted we also attempted to evaluate the relative effectiveness of the intervention of interest in LMICs versus HICs and gender-specific impacts. We summarized the evidence by outcome, including qualitative assessments of study quality and quantitative measures, according to the standard guidelines. A grade of “high,” “moderate,” “low,” and “very low” was used for grading the overall evidence indicating the strength of an effect on specific health outcome according to the Grading of Recommendations Assessment, Development and Evaluation (GRADE) criteria [20]. The GRADE approach specifically assesses methodological flaws within the component studies, consistency of results across different studies, generalizability of research results to the wider patient base, and how effective the treatments have been shown to be [21]. For purposes of systematic reviews, the GRADE approach defines the quality of a body of evidence as the extent to which one can be confident that an estimate of effect or association is close to the quantity of specific interest. Quality of a body of evidence involves consideration of within-study risk of bias (methodological quality), directness of evidence, heterogeneity, precision of effect estimates, and risk of publication bias [21].

Table 2 depicts the interventions for which we conducted an overview of systematic reviews, updated the existing reviews, and conducted de novo reviews, respectively.

### Strengths and limitations of the methodological approach

We have adopted three approaches for this review: (1) conducting an overview of systematic reviews; (2) updating existing reviews; and (3) conducting de novo reviews. Although an overview of systematic reviews builds on the conclusions of rigorous reviews of multiple quality intervention studies in different settings, avoids duplication of work, and allows for a much faster review, there are some potential limitations. The interventions on which primary data exist, but which have not been covered by a systematic review, will not have been included. Furthermore, an overview of systematic reviews relies on review authors' characterizations of the findings rather than on individual studies and therefore may be affected by selective reporting biases. It also misses upon studies not taken up by included reviews. However, we have quality rated the existing reviews for more transparency and conducted de novo reviews following a rigorous methodology. Furthermore, de novo reviews had to be conducted for interventions including sexual and reproductive health, micronutrient, and balance energy protein supplementation, nutrition interventions for pregnant adolescents, adolescent immunization, substance abuse, accidents and injury prevention, and adolescents living with chronic diseases since either no reviews existed or the existing reviews did not focus on the prespecified adolescent age group.

This introductory article helps understand the background, rationale, and methodology along with the conceptual framework which has consolidated the work in discussion for the following articles. With this understanding of the conceptual framework and methodology, we aimed to evaluate the effectiveness of interventions for improving adolescent health through existing systematic reviews and de novo reviews in the following articles. This article is followed by the following seven articles:

• Improving Adolescent Sexual and Reproductive Health: A Systematic Review of Potential Interventions: This review assessed the impact of interventions to improve sexual and reproductive health, preventing teenage pregnancy, preventing female genital mutilation/cutting, and dating violence prevention [42].
• Interventions to Improve Adolescent Nutrition: A Systematic Review and Meta-Analysis: This review evaluated the interventions to promote nutrition and prevent obesity, micronutrient and balanced energy protein supplementation, and nutrition interventions for pregnant adolescents [43].
• Systematic Review and Meta-Analysis of Interventions to Improve Access and Coverage of Adolescent Immunizations: This review assessed the effectiveness of interventions to improve immunization coverage for human papillomavirus measles, MMR, varicella, rubella, and TDap among adolescents [44].
• Interventions for Adolescent Mental Health: An Overview of Systematic Reviews: This overview of reviews assessed the effectiveness of interventions for eating disorders, violence, anxiety, depression, and suicide and suicidal behavior among adolescents and youth [45].
• Interventions for Adolescent Substance Abuse: An Overview of Systematic Reviews: This overview of reviews assessed the effectiveness of interventions to prevent substance abuse among adolescent population [46].
• Interventions to Prevent Unintentional Injuries Among Adolescents: A Systematic Review and Meta-Analysis: This article reviewed evidence for interventions to prevent unintentional injuries among adolescents [47].
• Adolescent Health Interventions: Conclusions, Evidence Gaps, and Research Priorities: This concluding article of the series summarized findings of all the previous articles in the series and relevance of the evidence for action in the post-2015 Millennium Development Goals era along with evidence gaps and future research priorities [48].

The core concept of this series of articles is to identify a set of interventions and delivery platforms which would help prioritize areas of action for the global community and individual countries alike, where they can focus on improving adolescent health and reduce mortality in the adolescent period. This would also identify the gaps in literature and areas where more evidence would be required. This would build on the increasing focus on improving adolescent health and well-being and support the achievement of women's, children's, and adolescents' health-related post-2015 new global framework of Sustainable Development Goals.

## Figures and Tables

**Figure 1:**
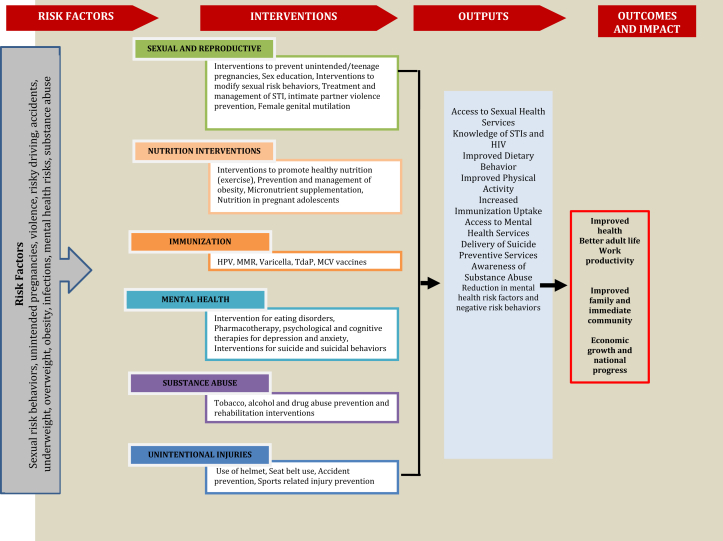
Conceptual framework. HPV = human papillomavirus; MCV = meningococcal vaccine; MMR = measles, mumps, and rubella; STI = sexually transmitted infection.

**Table 1:** Assessing methodological quality of systematic reviews (AMSTAR) criteria

1. Was a priori design provided? The research question and inclusion criteria should be established before the conduct of the review. 2. Was there duplicate study selection and data extraction? There should be at least two independent data extractors and a consensus procedure for disagreements should be in place. 3. Was a comprehensive literature search performed? At least two electronic sources should be searched. The report must include years and databases used (e.g., Central, Embase, and Medline). Keywords and/or MeSH terms must be stated, and where feasible the search strategy should be provided. All searches should be supplemented by consulting current contents, reviews, textbooks, specialized registers, or experts in the particular field of study and by reviewing the references in the studies found. 4. Was the status of publication (i.e., gray literature) used as an inclusion criterion? The authors should state that they searched for reports regardless of their publication type. The authors should state whether or not they excluded any reports (from the systematic review), based on their publication status, language, etc. 5. Was a list of studies (included and excluded) provided? A list of included and excluded studies should be provided. 6. Were the characteristics of the included studies provided? In an aggregated form such as a table, data from the original studies should be provided on the participants, interventions, and outcomes. The ranges of characteristics in all the studies analyzed, for example, age, race, sex, relevant socioeconomic data, disease status, duration, severity, or other diseases should be reported 7. Was the scientific quality of the included studies assessed and documented? A priori methods of assessment should be provided (e.g., for effectiveness studies if the author[s] chose to include only randomized, double-blind, placebo-controlled studies, or allocation concealment as inclusion criteria); for other types of studies alternative items will be relevant. 8. Was the scientific quality of the included studies used appropriately in formulating conclusions? The results of the methodological rigor and scientific quality should be considered in the analysis and the conclusions of the review, and explicitly stated in formulating recommendations 9. Were the methods used to combine the findings of studies appropriate? For the pooled results, a test should be done to ensure the studies were combinable, to assess their homogeneity (i.e., chi-square test for homogeneity, *I*^2^). If heterogeneity exists, a random effects model should be used and/or the clinical appropriateness of combining should be taken into consideration (i.e., is it sensible to combine?) 10. Was the likelihood of publication bias assessed? An assessment of publication bias should include a combination of graphical aids (e.g., funnel plot, other available tests) and/or statistical tests (e.g., Egger regression test). 11. Was the conflict of interest included? Potential sources of support should be clearly acknowledged in both the systematic review and the included studies.

MeSH = medical subject heading.

**Table 2:** The approach taken to review individual interventions (overview of existing reviews, updating existing review, de novo review)

Overview of reviews	Updated reviews	De novo reviews
- Interventions to prevent intimate partner violence - Interventions for prevention and rehabilitation of substance abuse - Interventions for adolescent mental health	- Interventions for preventing female genital mutilation/cutting - Interventions to promote healthy nutrition and prevent obesity - Interventions for eating disorders	- Interventions to improve sexual and reproductive health and prevent teenage pregnancy - Micronutrient and balance energy protein (BEP) supplementation among adolescents - Nutrition interventions for pregnant adolescents - Interventions for improving immunization coverage among adolescents - Interventions for accidents and injury prevention
